# Curiosity saved the cat: socio-emotional skills mediate the relationship between parental support and career exploration

**DOI:** 10.3389/fpsyg.2023.1195534

**Published:** 2023-09-26

**Authors:** Vítor Gamboa, Suzi Rodrigues, Filipa Bértolo, Beatriz Marcelo, Olímpio Paixão

**Affiliations:** ^1^Faculty of Human and Social Sciences, University of Algarve, Faro, Portugal; ^2^Research Center for Psychological Science, University of Lisbon, Lisbon, Portugal

**Keywords:** career exploration, parental support, curiosity, socio-emotional, skills mediation analysis

## Abstract

According to career literature, greater parental support seems to be associated with higher levels of career exploration. This relationship may be mediated by self-regulatory processes, such as social–emotional skills, as curiosity. However, despite the large number of empirical studies that analyze the antecedents of career exploration, there are no references, to our knowledge, to the role of socio-emotional skills. Following this gap, the present study aims to examine the extent to which perceived parental support is associated with career exploration through the mediating effect of curiosity (socio-emotional skill), among a group of 8th and 9th grade students from public schools in southern Portugal (*N* = 540). An integrated model was conducted using AMOS 20.0 and the results revealed that curiosity is a partial mediator of the relationship between perceived parental support and career exploration. These results highlight the importance of considering socio-emotional skills (such as curiosity) when designing interventions to foster adaptive career behaviors. Theoretical and practical implications are discussed to open the opportunity to progressively extend the participation of proximal contexts (e.g., families) to career and socio-emotional skills development processes.

## 1. Introduction

In the Portuguese educational system, the choice of the secondary education course is an important decision in which 8th and 9th grades students are involved. This career decision can be extremely challenging and can have medium and long-term effects on the individuals’ life courses (Germeijs and Verschueren, 2007; Saka and Gati, 2007). Despite not being a homogeneous group on how they solve vocational tasks, adolescents must explore different training paths and reflect about their interests, aspirations, and personal projects (Gamboa et al., 2014; Paixão and Gamboa, 2017). According to literature, there are two main reasons on why the career decisions that 8th and 9th grade students are involved can be so demanding: 1) the possible inexperience in making career decisions and the absence of objective criteria to support a good decision; and 2) the increasing complexity that characterizes the occupational world (Savickas, 2005; Blustein, 2006). In addition, these decisions may also be accompanied by high levels of anxiety, which may require the security and structure that is provided by parental support (Kenny and Medvide, 2013; Katz et al., 2018). In fact, parental support has been widely considered as being crucial to develop career adaptive behaviors (e.g., career exploration) (e.g., Whiston and Keller, 2004; Hartung et al., 2005; Kenny and Medvide, 2013; Ahn et al., 2022). Some empirical studies that have analyzed this relationship suggest that greater parental support is associated with higher levels of career exploration (Turan et al., 2014; Guan et al., 2015), and lower levels of indecision (Guay et al., 2003).

According to the career decision-making literature, this relationship may be mediated and moderated by self-regulatory processes, as will be the case of socio-emotional skills (e.g., Young et al., 1997; Blustein and Flum, 1999; Guay et al., 2003; Saka and Gati, 2007; Lent et al., 2016; Lipshits-Braziler et al., 2016; Paixão and Gamboa, 2022). Socio-emotional skills can be defined as the ability to regulate thoughts, emotions, and behaviors, and can be developed throughout life, through formal and informal learning experiences, in school and family contexts, for example (Kankaras and Suarez-Alvarez, 2019). It is during adolescence that we can observe a significant development of these types of self-regulatory skills (e.g., tolerance and curiosity), which can play a crucial role in how students deal with vocational tasks during the transition to secondary education (Chernyshenko et al., 2018). According to Howard and Ferrari (2021), the growing interest in the relationship between socio-emotional skills and career development stems from the idea that managing emotions facilitates exploration and progress in career decision-making. Thus, in complex tasks such as choosing a secondary school course, socio-emotional skills are pivotal in the way each student deals with the stress and ambiguity often associated with career exploration. In other words, students with higher levels of socioemotional skills are less likely to drop out when facing difficulties during career exploration activities (Di Fabio et al., 2012; Di Fabio and Kenny, 2015). The socio-emotional skill of curiosity, which is anchored in openness to experience (OECD, 2021), is defined as the interest in learning and exploring the unknown, and in taking risks. Curiosity may relate to information seeking behaviors associated to the self and occupational contexts. Thus, in our approach to the study of the influence of socioemotional skills we chose to focus on curiosity, as it assumes a crucial role in career exploration and in the most established career theories (e.g., Career Construction Theory, Savickas, 2005). Therefore, the main objective of this study was to analyze the relationship between parental support and career exploration, considering the mediating effect of an important socio-emotional skill: curiosity.

### 1.1. Career exploration

Career exploration is a complex psychological process of exploration of the self and the external environment (Jordaan, 1963; Patton and Porfeli, 2007; Porfeli and Lee, 2012) that ensures career adaptability (Blustein, 1997; Savickas, 2005; Savickas et al., 2009) and has a particular significance in transitional periods (e.g., basic to secondary education school transition) in which individuals are frequently challenged with new roles (Kalakoski and Nurmi, 1998; Flum and Blustein, 2000; Pryor and Bright, 2011). According to Super et al. (1996), during the life stage of exploration, adolescents’ main tasks include narrowing occupational choices, formulating career goals, and implementing career plans. More recently, in career self-management model (CSM, Lent and Brown, 2013; Lent et al., 2016), it is hypothesized that exploratory actions contribute directly to various career outcomes, such as career decidedness or perceived employability (e.g., Kleine et al., 2021). Also, according to this model, the exercise of adaptive career behaviors (e.g., engaging in career exploration) is assumed to be affected (directly and indirectly) by the individual cognitive variables (e.g., career goals, expectations) and environmental supports and barriers. Highlighting the role of self-management in the career exploration process, the CSM model (Lent and Brown, 2013) aims to explain the conditions that predict the use of adaptive career behaviors (such as career exploration), that individuals use to manage their career development and to cope with career-related challenges (Jiang et al., 2019).

For Career Construction Theory (CCT, Savickas, 2013), career exploration is considered a coping behavior in the structural model of career adaptability. Here, adaptability resources (e.g., curiosity) may play a self-regulator role that can enable the individual to explore relevant career information (coping behavior) and consequently adapt and cope with expected and unexpected career transitions. On the other hand, career exploration is also described as a process that integrates self-determination mechanisms (e.g., socio-emotional skills), into career development (Blustein, 1988; Flum and Blustein, 2000; Flum, 2015). Thus, by considering socioemotional skills as antecedents of career exploration we can better understand how young people regulate their behaviors as they incorporate information from the world around them and evaluate their learning and occupational life experiences. In sum, career exploration emerges as a critical ingredient in adolescents’ career development as it raises individuals’ awareness of their career options and how their interests, values and aspirations relate with the world of work (Jiang et al., 2019). Additionally, empirical evidence suggests that career exploration promotes coherent career plans and facilitates the career decision-making process (Creed et al., 2007; Patton and Porfeli, 2007; Kleine et al., 2021; Paixão and Gamboa, 2022).

### 1.2. Parental support as a contextual antecedent of career exploration

Career literature has consistently highlighted the importance of family in career development on a wide range of vocational processes, such as career exploration (e.g., Whiston and Keller, 2004; Hartung et al., 2005; Kenny and Medvide, 2013). Since career exploration requires a certain openness to the unknown and high levels of self-confidence, we can expect that the quality of parent–child relationships may reduce anxiety and stimulate the search for useful career information. Anchored in developmental-contextual (e.g., Vondracek et al., 1986; Young et al., 2002), relational (e.g., Blustein, 2011; Flum, 2015; Kenny et al., 2018) and social cognitive career models and theories (e.g., Lent et al., 1994), empirical research suggest that parental support, in its multiple dimensions (e.g., emotional support, instrumental support), is associated with higher levels of exploration (e.g., Dietrich et al., 2011; Guan et al., 2015; Estreia et al., 2018; Maftei et al., 2023). Several authors have suggested that the quality of support received in proximal contexts (e.g., parents, teachers, peers) can influence individuals’ career exploration (e.g., Whiston and Keller, 2004; Reeve, 2009; Lent and Brown, 2013; Turan et al., 2014; Guan et al., 2015; Rodrigues et al., 2017; Ryan and Deci, 2019). Dietrich and Kracke (2009) and Dietrich et al. (2011) reported that parental figures are the main source of support considered by adolescents when approaching academic transitions. Specifically, the perception of parents’ interest and involvement seems to lead to an increase in the individuals’ levels of career exploration. Similar results were found by Guan et al. (2015), in a sample of Chinese university students. Using Social Cognitive Career Theory (Lent et al., 1994), Ginevra et al. (2015) found that both mothers’ and fathers’ perceptions of support predicted their adolescents’ career choice through the mediating effect of the youths’ perceptions of parental support. More recently, Estreia et al. (2018) concluded that father emotional support predicts environmental exploration while mother emotional support predicts self-exploration. Taken together, these results underline that perceived parental support has a significant impact on career exploration.

### 1.3. Parental support as a contextual antecedent of curiosity

As previously mentioned, parents play a central role in career development, as they can foster adolescents’ agency in career exploration and decision-making, through multiple shared activities. While the impact of parental support on motivation has been extensively studied (e.g., Katz et al., 2018; Paixão and Gamboa, 2022), it appears that the relation between parental support and development of socio-emotional skills has been less explored (e.g., Di Fabio and Kenny, 2015; Howard and Ferrari, 2021). Socio-emotional skills encompass the ability to recognize and regulate emotions (e.g., Kankaras and Suarez-Alvarez, 2019) and it is through observation and interaction with parents that crucial self-regulatory skills, such as self-efficacy, goal setting ability and socio-emotional skills are often acquired (e.g., Young et al., 1997, 2002). Specifically, it is in the proximal relational contexts, such as the family, that youngsters will progressively develop not only their ability to negotiate and manage conflicts, but also a set of self-regulatory skills, namely and among others: persistence, goal orientation, and curiosity. Therefore, it is important to analyze the extent to which the different facets of parental support can influence the development of curiosity in adolescents.

Considering the strong connection observed between motivation and emotion in explaining behavior across distinct achievement contexts (e.g., Reeve, 2009), it seems reasonable to argue that the same contextual factors that influence motivational processes could also explain the development of socio-emotional skills. Using the conceptual framework of the Self-Determination Theory (SDT, Deci and Ryan, 2000), several empirical studies offer evidence of the impact of parental support on motivation (e.g., Guay et al., 2003; Rodrigues et al., 2017; Katz et al., 2018). Overall, these studies consistently demonstrate that parental support is associated to more self-determined types of motivation and with better performance outcomes, particularly when it fosters autonomy. Conversely, if parental support tends to be characterized by control or lack of involvement, is often associated to less self-determined types of motivation and to poorer performance.

As a socio-emotional skill, curiosity is associated with observation, hypothesis testing, engaging in new learning activities, taking risks, and having a clear and positive orientation towards the future. Consequently, curiosity also improves learning outcomes and provides intrinsic incentives for lifelong self-development (Chernyshenko et al., 2018). In other words, curiosity is associated to the adolescents’ openness to new learning experiences and their tolerance to ambiguous and unfamiliar information. Thus, it is expected that strong and secure relationships with parental figures could foster the necessary sense of security and autonomy to explore the unknown. Moreover, it is also assumed that more instrumental parental support (e.g., guidance on “how to do”) will facilitate the development of the skills needed to explore and organize the information gathered from distinct sources.

### 1.4. Curiosity as individual antecedent of career exploration

Based on what was mentioned before, we can conclude that the adjustment to a secondary school course (10th, 11th, and 12th grades) will depend, to some extent, on the quality of the career exploration carried out by the student during basic education (e.g., 8th and 9th grades). This is what makes the career exploration process widely considered as a critical ingredient in the career development of adolescents (e.g., Blustein, 1997; Patton and Porfeli, 2007), especially when considering the career outcomes, it can predict (e.g., vocational identity, adjustment to learning contexts, commitment to career choices). Therefore, it is important to study not only the effect of contextual predictors of exploration, but also the effect of its individual antecedents (Jiang et al., 2019). To this end, we follow the suggestion of authors who advocate for the study of variables related to motivation and emotion in explaining adaptive career behaviors (e.g., Blustein, 1988; Guay et al., 2003; Paixão, 2008; Lent et al., 2016). Indeed, in intentional and goal-directed actions, such as career exploration activities, emotions may have a self-regulatory function in information-seeking behaviors (Young et al., 2002). In this regard, it should be noted that the results observed in the studies by Lee et al. (2016) and, more recently, Gamboa et al. (2021) corroborate the assumptions of Blustein (1988) and Flum and Blustein (2000), who describe exploration as a goal-oriented process. Thus, research is needed to analyze the role of the processes that initiate, cease, sustain, and guide career exploration. In their systematic review, Jiang et al. (2019) identify a set of motivational variables (e.g., goals, self-efficacy, expectations, and intrinsic motivation) and personality variables as individual antecedents of career exploration. Kleine et al. (2021), in a meta-analysis, based on the career self-management model of Lent and Brown (2013), concluded that social cognitive variables and some personality traits (e.g., openness to experience, extraversion, and conscientiousness) are positively associated with career exploration behaviors. Nevertheless, despite the large number of empirical studies analyzed, the works of Jiang et al. (2019) and Kleine et al. (2021) do not make explicit references to the role of socio-emotional skills in career exploration. Generally, there are few studies analyzing the role of social and emotional skills in career development, particularly concerning career exploration (Kidd, 2004; Hartung, 2010, 2011). However, research results have been supporting the association between socio-emotional skills and career behavior. For example, in a study that included 14 parent–child dyads, Young et al. (1997) found evidence of the regulatory role of emotions in the joint construction of career projects. Farnia et al. (2018) observed a significant effect of emotions (positive and negative) on levels of indecisiveness in a sample of university students. Also, very recently, Santos et al. (2018) found a negative association between lack of occupational information and emotional regulation strategies. Regarding the socio-emotional skill curiosity, it seems to be associated with openness to experience, interest in novelty, and a desire to learn new things. In the conceptual model adopted by the OECD (2021), curiosity relates to openness to experience (Big Five), which is positively correlated with career exploration (particularly self-exploration) (Lee et al., 2016). Curiosity also assumes a central role in individual agency variables being associated to self-determined types of motivation (e.g., Deci and Ryan, 2000; Flum and Blustein, 2000), affecting not only the quantity but also the quality of career exploration (Paixão and Gamboa, 2017, 2022).

In career psychology, curiosity is frequently positioned as an adaptability resource, following the assumptions of CCT (e.g., Savickas and Porfeli, 2012; Savickas, 2013), which means that this concept can be defined as a self-regulatory resource related to the willingness to explore the environment, aiming to acquire information about the self and the outside world (Hirschi et al., 2015). According to Savickas (2013), adaptability resources (attitudes, beliefs, and competencies) refer to the psychosocial strengths that affect self-regulation when coping with career tasks and transitions. Therefore, the adaptive individual is conceptualized as someone that shows curiosity for exploring possible selves and future scenarios (Savickas, 2013). Consequently, career curiosity favors the organization of information that is considered useful to career decision-making and to the adjustment to new learning contexts. For this reason, we can assume that career curiosity prevents unrealism about the world of work as well as inaccurate images of the self. Previous research has shown results that support this. For example, Hirschi et al. (2015) found a positive and significant association between curiosity and career exploration, and Li et al. (2015) showed results of regression analysis that present curiosity as a significant predictor of career exploration. In summary, we can infer that curiosity is positively associated with career exploration.

### 1.5. Present study and hypothesis

As we have explained before, curiosity can be considered as a malleable construct (Di Fabio and Kenny, 2015; Chernyshenko et al., 2018) suggesting that it may be developed through a set of activities within the family context, such as parent-adolescent career conversations (e.g., Young et al., 1997). Additionally, Self Determination Theory (SDT, Deci and Ryan, 2000) and relational and socio-cognitive approaches to career development (e.g., Young et al., 1996; Blustein and Flum, 1999; Blustein, 2011) advocate that the support provided by the family will help in reducing anxiety and will stimulate the search for new learning experiences (e.g., curiosity). Howard and Ferrari (2021) also support the idea that the development of socio-emotional skills would both support and be supported by the development of competencies in the career domain. Therefore, we can assume that curiosity is a significant component in the relationship between parental support and career exploration.

The first aim of this study was to analyze the relationship between career exploration (self-exploration and environmental exploration) and parental support (instrumental assistance, verbal encouragement, career related modeling, emotional support), and curiosity, respectively. Based on the literature review presented before, we expect to find positive correlations between parental support and career exploration (*H1*), positive correlations between career exploration and curiosity (*H2*), and positive correlations between parental support and curiosity (*H3*). Considering unidirectional links, we expect to find positive influence of parental support on curiosity (*H4*), positive influence of parental support on career exploration (*H5*), and positive influence of curiosity on career exploration (*H6*). Finally, we expect that curiosity (as a socio-emotional skill as a self-regulatory process) can have a mediation role on the relationship between parental support (contextual factors) and career exploration (career adaptative behaviors).

## 2. Method

### 2.1. Participants and data collection

We used a sample of 540 students, distributed among 8th (*N* = 328; 60.7%) and 9th grades (*N* = 212; 39.3%), from public schools in southern Portugal. The sample comprises 273 boys (50.6%) and 267 girls (49.4%), aged between 13 and 15 years old (M = 13,72, SD = 0.69). After an initial phase where the study was presented to schools, appropriate informed consent procedures were followed in collecting data including obtaining parents’ and school boards’ permissions. The administration of the instruments was made by trained coresearchers in a classroom context, with the assistance of the school psychologist. On average, each assessment lasted 20 min. Before starting to fill out the questionnaire, participants were informed about the general topic of the study, the voluntarily character of their participation and confidentiality of their answers was assured.

### 2.2. Measures

Parental support was assessed with the *Career-Related Parent Support Scale* (CRPSS, Turner et al., 2003; adapt. Gamboa et al., 2021). The CRPSS aims to assess students’ perceptions of parental support toward career and educational development along the four sources of self-efficacy expectations proposed by Bandura (1997). This 27 items scale consists of four subscales: 1) Instrumental Support (6 items, e.g., “My parents help me to choose out of school activities that may be useful in my future professional career”, 2) Career Modeling (7 items, e.g., “My parents have already shown me where they work”), 3) Verbal Persuasion (5 items, e.g., “My parents praised me for doing my schoolwork well”), and 4) Emotional Support (6 items, e.g., “My parents say they are proud of me when I am successful in school”). Items were rated using a 5-point Likert-type scale, ranging from 1 (strongly disagree) to 5 (strongly agree). The higher the score is, the greater the perceived parental support. The validity and reliability of the scale have already been demonstrated, both in the original version (Turner et al., 2003) and in the Portuguese version (Gamboa et al., 2021). In the present study, the estimates of internal consistency in the subscales varied between 0.81 (Verbal Encouragement) and 0.87 (Emotional Support) and for the entire scale, the value observed was 0.93.

Career exploration was assessed using the Portuguese version of the Career Exploration Survey (CES; Stumpf et al., 1983; adapt. Taveira, 1997). The CES is a multidimensional self-administered scale with 54 items (Likert-type response format), designed to assess beliefs, processes, and reactions to career exploration. In the present study, we only used the items that compose two processes of career exploration: Self-Exploration (5 items, e.g., “In the last 3 months I reflected on how my past integrates with my future career”) and Environmental Exploration (4 items, e.g., “In the last 3 months I went to various career orientation programs”). The validity, reliability, and multidimensionality of the CES have been widely demonstrated in its’ different versions. Cronbach’s alpha for the Portuguese version ranged from 0.63 to 0.83. Research that used this version found internal consistency values for the two exploration processes used in this study between 0.74 and 0.79 (Paixão and Gamboa, 2017, 2022; Gamboa et al., 2021) and we obtained values of 0.80 for environmental exploration and 0.76 for self-exploration.

Socio-emotional skills were assessed with the Portuguese version of Socio-Emotional Skills Survey (SSES; OECD, 2021), provided by Calouste Gulbenkian Foundation. This survey aims to better understand students’ contextual factors (e.g., school, home, community) and characteristics that directly or indirectly influence the development of social and emotional skills. The SSES conceptual framework is based on the OCDE framework (Chernyshenko et al., 2018; Kankaras and Suarez-Alvarez, 2019) and was developed in reference to the ‘Big Five Model’ (John et al., 2008) that distinguishes 15 skills in five dimensions: 1) Task Performance (self-control, responsibility, persistence), 2) Emotional Regulation (stress resistance, optimism, emotional control), 3) Collaboration (empathy, trust, cooperation), 4) Open-Mindedness (tolerance, curiosity, creativity), 5) Engaging with Others (sociability, assertiveness, energy). In this study, we used Curiosity (from Open-Mindedness dimension) as a skill that represents an interest in ideas and love for learning, understanding and for intellectual exploration. This skill was measured using eight items (e.g., “I like to know how things work”) rated on a 5-point Likert-scale. The validity and reliability of the scale have been demonstrated in other studies, reporting Cronbach’s alpha that ranged from 0.80 and 0.81 (e.g., Kankaras et al., 2019; Salmela-Aro and Upadyaya, 2020), which is aligned with the value that we found in this study (*α* = 0.81).

### 2.3. Analysis

In the first step, we computed the means, standard deviations, and correlations among the variables. Secondly, Path analysis was performed in AMOS 28.0 (Amos Development Corporation, FL) with maximum likelihood estimation, to test whether the career related parent support (emotional support, instrumental assistance, career-related modeling, and verbal encouragement) influenced directly and indirectly environmental exploration and self-exploration, through the mediating effect of curiosity. Mediation analysis is a statistical procedure to elucidate the mechanism that intervenes between the independent and outcome variables. It explains how the independent variable, through the intermediate variable or mediator, affects the dependent variable. We assessed significant indirect effects by computing bias-corrected bootstrap intervals in AMOS 28.0 bootstrapping function (2,000 samples) at 95% confidence intervals (CIs95; Preacher and Hayes, 2008). Here, if the CIs 95 of the indirect effect does not include zero, we can consider the existence of mediation effects. Goodness of fit was judged according to the following fit indices: χ^2^/*df* ratio (<3), standardized root mean squared residual (SRMR <0.08), the comparative fit index (CFI > 0.90), the normed fit index (NFI > 0.90), and the root mean square error of approximation (RMSEA <0.08, 95% confidence interval lower and upper limits, hereafter 95% CI [LL, UL]) (Byrne, 2016; Kline, 2016).

## 3. Results

Table 1 shows means, standard deviations, bivariate correlations, and Cronbach Alphas for the variables in study. The four dimensions of the CRPSS were positively correlated with environmental exploration, self-exploration, and curiosity, being the most highlighted values those observed between Emotional Support and Self-Exploration (*r* = 0.47, *p* < 0.01) and between Emotional Support and Curiosity (*r* = 0.44, *p* < 0.01). Additionally, Curiosity also presented positive correlation with Environmental Exploration (*r* = 0.25, *p* < 0.01) and Self-Exploration (*r* = 0.42, *p* < 0.01).

**Table 1:** Descriptive statistics, reliabilities, and correlations of variable (*N* = 540).

	M	SD	1.	2.	3.	4.	5.	6.	7.
1. EM	3.76	0.91	(0.87)						
2. IA	3.66	0.85	0.77^**^	(0.80)					
3. CRM	4.16	0.76	0.48^**^	0.50^**^	(0.83)				
4. VE	4.25	0.75	0.64^**^	0.62^**^	0.58^**^	(0.81)			
5. EE	2.87	1.04	0.37^**^	0.38^**^	0.26^**^	0.20^**^	(0.80)		
6. SE	3.27	0.93	0.47^**^	0.39^**^	0.21^**^	0.24^**^	0.57^**^	(0.76)	
7. Curiosity	3.93	0.61	0.44^**^	0.40^**^	0.33^**^	0.40^**^	0.25^**^	0.42^**^	(0.81)

** *p* < 0.01. Cronbach Alpha for each variable is presented between parentheses. EM, Emotional Support; IA, Instrumental Assistance; CRM, Career-Related; Modeling; VE, Verbal Encouragement; EE, Environmental Exploration; SE, Self-Exploration.

In the next step, seven *t* tests revealed significant gender differences only in Curiosity (*t* = 2.685, *p* < 0.01, *d* = 0.23). Specifically, girls (*M* = 4.00; *SD* = 0.59) perceived themselves as more curious than boys (*M* = 3.86; *SD* = 0.63). There was no sign of multicollinearity issues among career support variables as we obtained values of Variance Inflation Factors (VIF) < 10 and Tolerance values >0.10 (Kline, 2016). In the last step, we examined the fit indices for the proposed model, which illustrates the hypothesis in study, and it was not found to fit with the data adequately for all criteria, χ^2^(1, *N =* 540) = 133.15; RMSEA = 0.50; CFI = 0.92; NFI = 0.92; SRMR = 0.07. We analyzed modification indices and standardized residuals to verify any suggestions that could lead to an improvement to the model that also would conceptually make sense. Using a step-by-step approach and checking fit indices after respective modifications we eliminated nonsignificant direct effects (due to their low reliability), and covariate the residual errors of the exploration variables (Byrne, 2016).

**Figure 1:**
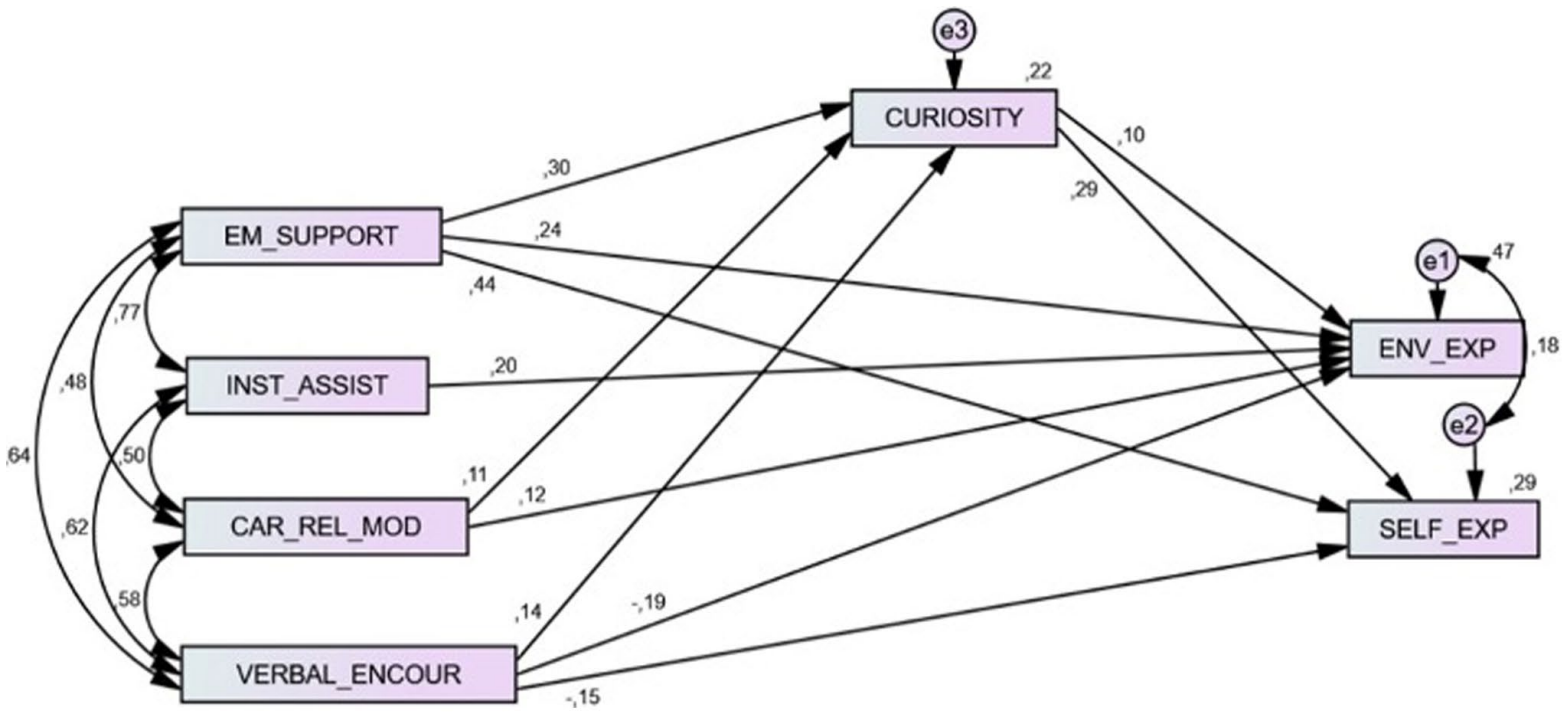
Final Model: χ^2^(3, *N* = 540) = 1.49; RMSEA = 0.03; CFI = 0.99; NFI = 0.99; SRMR = 0.01. EM_SUPPORT, Emotional Support; INST_ASSIST, Instrumental Assistance; CAR_REL_MOD, Career-Related Modeling; VERBAL_ENCOUR, Verbal Encouragement; ENV_EXP, Environmental Exploration; SELF_EXP, Self-Exploration.

The final model (Figure 1) shows good fit to data χ^2^(3, *N =* 540) = 1.49; RMSEA = 0.03; CFI = 0.99; NFI = 0.99; SRMR = 0.01.

The standardized coefficients revealed significant direct effects between Emotional Support and Curiosity (*β* =0.30, *p* < 0.01), Environmental Exploration (*β* =0.24, *p* < 0.01), and Self-exploration (*β* =0.44, *p* < 0.01). Instrumental Assistance emerges as having direct influence on Environmental Exploration (*β* =0.20, *p* < 0.01). Career-Related Modeling also shows significant values to Curiosity (*β* =0.12, *p* < 0.01) and Environmental Exploration (*β* =0.11, *p* < 0.05). The last career related support variable, Verbal Encouragement, seems to be positively associated to higher Curiosity (*β* =0.14, *p* < 0.01), Environmental Exploration (*β* = −0.19, *p* < 0.01) and Self-Exploration (*β* = −0.15, *p* < 0.01). Additionally, we observed that Curiosity has direct influence on Environmental Exploration (*β* =0.10, *p* < 0.05) as well as on Self-Exploration (*β* =0.29, *p* < 0.01).

We assessed significant indirect effects by computing bias-corrected bootstrap intervals in AMOS 28.0 bootstrapping function (2,000 samples) at 95% confidence intervals (CIs95; Preacher and Hayes, 2008). Here, if the CIs95 of the indirect effect does not include zero, we can consider the existence of mediation effects.

Table 2 summarizes the six significant indirect effects that were found. Curiosity partially mediated the effect of Emotional Support on Environmental Exploration (CI_95_ [0.001, 0.063], *β* = 0.03, *p* < 0.05) and Self-Exploration (CI_95_ [0.045, 0.135], *β* = 0.09, *p* < 0.01). Concerning the relationship between Career-Related Modeling and career exploration variables, Curiosity partially mediates the effect to Environmental-Exploration (CI_95_ [0.001, 0.031], *β* = 0.01, *p* < 0.05) and fully mediates the effect to Self-Exploration (CI_95_ [0.004, 0.065], *β* = 0.03, *p* < 0.05). Finally, results show partial mediation of Curiosity on the effect of Verbal Encouragement on Environmental Exploration is (CI_95_ [0.001, 0.041], *β* = 0.01, *p* < 0.05) and on Self-Exploration (CI_95_ [0.007, 0.081], *β* = 0.04, *p* < 0.05) was also partially mediated by Curiosity.

**Table 2:** Bias-corrected bootstrapping test of the mediation effect of curiosity.

Independent Var.	Mediator Var.	Dependent Var.	Estimate	95% Conf. Int.
Emotional support	Curiosity	Environm. Exp.	0.03^*^	[0.001, 0.063]
		Self-Exploration	0.09^**^	[0.045, 0.135]
Career-related modeling		Environm. Exp.	0.01^*^	[0.001, 0.031]
		Self-Exploration	0.03^*^	[0.004, 0.065]
Verbal encouragement		Environm. Exp.	0.01^*^	[0.001, 0.041]
		Self-Exploration	0.04^*^	[0.007, 0.081]

*N* = 540. 95% confidence interval does not include zero.

*p < 0.05; **p < 0.01.

Globally, the direct and indirect effects found in the final model explain 29% of the variance of self-exploration and 18% of the variance of environmental exploration.

## 4. Discussion

The purpose of this study was to analyze how career-related parental support associates to career exploration behaviors (self and environmental) and in which degree curiosity, as a self-regulatory process, mediated the effect of parental support on career exploration, among a group of 8^th^ and 9^th^ grade students. To address our goals, we tested an integrated model through path analysis using AMOS 20.0.

Our correlations analysis allows us to confirm *H1*, given that we can observe positive correlations between both types of career exploration and all dimensions of parental support. These results agree with the results of some empirical studies carried out in the last few years that support the importance of family on enhancing exploration behaviors (e.g., Dietrich et al., 2011; Guan et al., 2015; Estreia et al., 2018; Maftei et al., 2023), especially when approaching school transitions (Dietrich and Kracke, 2009; Dietrich et al., 2011). Our results also reinforce the assumptions of the relational perspectives of career exploration (e.g., Blustein, 1997; Blustein and Flum, 1999), i.e., the support provided by parents promotes adolescents’ openness to explore the different courses of secondary education and their feasibility.

Correlational analysis also shows positive associations between curiosity and both types of career exploration, helping to confirm *H2*. These results corroborate studies that support the association between social and emotional skills and career behavior (Young et al., 1997; Farnia et al., 2018; Santos et al., 2018). Meaning, as suggested by Howard and Ferrari (2021), that students’ ability to manage their emotions is crucial to their success in the transition and adjustment to secondary school. In addition, these results are also aligned with OECD (2021) framework that considers curiosity as an important socio-emotional skill related to openness to experience, which is often associated to career exploration behaviors (Lee et al., 2016; Kleine et al., 2021). Similarly, Hirschi et al. (2015) and Li et al. (2015) have shown that curiosity has a positive and significant relationship with career exploration, especially with self-exploration. In summary, and in agreement to relational and self-determination perspectives of career development (e.g., Blustein and Flum, 1999), we can conclude that the most curious students are also those who are most frequently involved in career exploration activities.

*H3* can also be confirmed since we can observe positive associations between all variables of parental support and curiosity. However, by analyzing the direct effects on our final model we can observe that, regarding the four types of parental support, affective and emotional behaviors seem to relate to curiosity more strongly than instrumental behaviors (e.g., instrumental assistance). These results help us to partially confirm *H4* and reinforce that the perceived emotional support provided by the parental figures might be determinant to develop curiosity towards career subjects (Young et al., 1996, 1997; Di Fabio and Kenny, 2015; Chernyshenko et al., 2018). Thus, considering that career decisions are not easy to make during adolescence, we can conclude that curiosity and confidence to explore the self and the world of work has its roots in the security and structure provided by parents.

The results of the final model show that some of the parental support variables positively associate with environmental exploration (emotional support, instrumental assistance, and career-related modeling) and self-exploration (emotional support), but contrary to what we usually find in literature, it suggests a negative effect of verbal encouragement on these two types of exploration. These results allow us to partially confirm *H5*. This effect may be related to the nature and content of the items that compose this dimension of perceived parental support (e.g., item – *my parents encourage me to make good grades*). That is, these students may associate verbal encouragement with greater control and interference from their parental figures (e.g., Boerchi and Tagliabue, 2018) resulting in some inhibition or avoidance to exploration behaviors. In this regard, other studies have expanded the discussion of how important the quality of support in career behaviors can be, stating that a control type of support might be detrimental to adaptive career outcomes (Reeve, 2009; Lent and Brown, 2013; Ryan and Deci, 2019; Paixão and Gamboa, 2022). In addition, instrumental assistance and career-related modeling only associates with environmental exploration. In other words, according to our results, while emotional support helps to answer the question “*Who am I?”* (self-exploration), the more instrumental modalities of support help to answer the question *“What do I want or need?”* (Environmental exploration). From a career development viewpoint, we should underscore that career-related parental support does not have the same effect on the two dimensions of career exploration, as observed in the studies conducted by Estreia et al. (2018) and Rodrigues et al. (2017).

*H6* can also be confirmed by the results of the final model. As theoretically expected, curiosity seems to play an important self-regulatory role in adolescents’ career transitions (Savickas, 2013). This means that developing this socio-emotional skill can lead to higher levels of career exploration, facilitating the career-decision making process (Creed et al., 2007; Patton and Porfeli, 2007; Kleine et al., 2021; Paixão and Gamboa, 2022).

Lastly, the results partially confirm *H7*, since that curiosity shows up as partial mediator of the relationship between emotional support, career-related modeling and verbal encouragement and environmental exploration and self-exploration. These results are aligned with the career decision-making literature that suggest a relationship between parental support and career adaptive behaviors (e.g., career exploration) and highlight that this relation might be mediated and moderated by self-regulatory processes (e.g., Young et al., 1997; Blustein and Flum, 1999; Guay et al., 2003; Saka and Gati, 2007; Lent et al., 2016; Lipshits-Braziler et al., 2016; Paixão and Gamboa, 2022). In our final model, we can also observe that curiosity fully mediated the effect of career-related modeling on self-exploration. From our point of view, this result underlines the role of curiosity in more distal strategies of information gathering, as will be the case of career-related modeling (e.g., my parents have taken me to their work). In contrast to more proximal strategies, these distal strategies promote greater complexity and cognitive integration in the process of career exploration. Therefore, we can consider that curiosity can, in fact, constitute a main mechanism in the construction of the new meanings (self-exploration) that result from the learning experiences that take place in family and work contexts.

As in previous studies that analyzed the antecedents of career exploration (e.g., Blustein, 1988, 1997; Patton and Porfeli, 2007; Rodrigues et al., 2017; Maftei et al., 2023), in our model the explained variance of self-exploration and environmental exploration is relatively modest. An analysis that included three variables related to parental support and dispositional optimism, the model tested by Maftei et al. (2023) explained 25% of the career exploration variance. In turn, in the study of Guan et al. (2015), with a sample of Chinese university students, parental support explained about 19% of the variance of career exploration, after controlling for the effects of a wide range of sociodemographic variables. Already in 1997, Blustein hypothesized the existence of other factors associated with career exploration that had not yet been properly identified, mainly those of cognitive-motivational nature. In the present study, we expanded the set of variables capable of explaining exploration behaviors, including socio-emotional skills. We only included curiosity, due to the centrality of this skill in the career literature, leaving out other competences that are theoretically relevant in explaining exploration behaviors. Therefore, in future research, the inclusion of a greater number of socio-emotional skills (e.g., adaptability, empathy, assertiveness, optimism, tolerance, responsibility) may increase the explained variance of career exploration behaviors.

## 5. Theoretical and practical implications

This study offers an important contribution to career literature by showing evidence of the effects of socio-emotional skills, specifically curiosity, on the career exploration behaviors. In fact, despite the relationship between these variables, very few empirical studies present explicit support to these effects. In career domain, socio emotional skills have been widely investigated with an apparent lack of career theoretical frameworks (e.g., Howard and Ferrari, 2021). Our results follow the holistic theories that preconize approaches that integrate simultaneously emotional and career processes (e.g., Young et al., 1996; Hartung, 2011). The tested model helps us to better understand the beneficial effects of socio-emotional skills in career behaviors (e.g., exploration behaviors) and thus contributes to a more integrated view of the relationships observed between emotions and career. Globally, the current article is in line with the substantial research literature that supports the relationship between parental support and career development outcomes (e.g., Ahn et al., 2022; Maftei et al., 2023). Our model incorporated four dimensions of parental support to better explain career exploration and curiosity in accordance with the main propositions of the socio cognitive theory (e.g., Bandura, 1997). Although significantly correlated among each other, in the tested model, the four dimensions of parental support are not identically associated (directly and indirectly) with curiosity and career exploration. Therefore, our results also reinforce the importance of conceptualizing parental support as a multidimensional construct. Also, the mediating effect of curiosity on the relationship between parental support and career exploration leads us to confirm the important role that this socio-emotional skill plays in regulating exploration behaviors and the adjustment to learning and work contexts. We can also draw theoretical implications from this mediation effect. On the one hand, it supports constructivist (e.g., Savickas, 2005) or socio cognitive (e.g., Lent et al., 1994) theories, which consider emotional variables as personal resources that favor agency, self-reflection, and purposeful involvement in vocational tasks. On the other hand, our model also suggests the consolidation of career education models aimed simultaneously at vocational and emotional processes (e.g., Young et al., 1997, 2002; Howard and Ferrari, 2021).

From the point of view of educational policies, the OECD report (2021) argues that in an increasingly turbulent world socio-emotional skills (e.g., curiosity and openness) should be the bedrock of students’ well-being and academic achievement. In this sense, we suggest the systematic and intentional incorporation of the development of these competences in the school curriculum.

Regarding practical implications, these findings suggest the importance of considering socio-emotional skills (such as curiosity) in career interventions. Also, it opens the opportunity to progressively extend the participation of proximal contexts (e.g., families) to career development processes, since that these skills can be: 1) enhanced by greater involvement of those who assume an important role on the individual’s career choices, especially in complex tasks and transitions (e.g., choosing a secondary school course); and 2) developed through formal and informal learning experiences (e.g., school, household context). In this sense, in addition to information about courses and professions (e.g., instrumental support), parents, teachers and school psychologists must provide the necessary emotional support for curiosity and self-exploration. Our results also suggest that the promotion of curiosity should be considered in career intervention, especially in the more distal modalities of occupational information gathering, for example in internships or job shadowing activities. This is because it is precisely in real work contexts that adolescents can more easily explore the different aspects of their selves, test new roles, and develop social and career skills.

The direct and indirect effects found in our final model show that the model has the capacity to better explain self-exploration (29% of variance explained) than environmental exploration (18% of variance explained). Despite these values not being too expressive, it broadens our knowledge about possible significant predictors of career exploration. For instance, this effort meets the gap that some authors have already identified in the literature (e.g., Blustein, 1988, 1997; Patton and Porfeli, 2007; Jiang et al., 2019; Kleine et al., 2021).

## 6. Limitations and future research

As our study is cross-sectional, we suggest that future research could use longitudinal designs that allows a more accurate analysis on the development and role of students’ socioemotional skills before and after entering secondary school. Additionally, in future research, following the conceptual framework adopted by the OECD, we should consider a broader range of socio-emotional skills and analyze them as mediating and moderating variables. That is, if socio-emotional skills refer to different processes (openness to experience, emotional control) we can expect different effects in career exploration. Considering the gender differences observed for the mediating variable (curiosity), this presents an opportunity to be taken into account and addressed in future research, for example, by studying the moderating role of gender in relations between the studied variables. Finally, a person-centered approach could be helpful to explore the role of socio-emotional skills on career exploration behaviors and on perceived parental support among distinct profiles.

## Data availability statement

The raw data supporting the conclusions of this article will be made available by the authors, without undue reservation.

## Author contributions

VG designed the study and wrote the first draft of the manuscript. VG, SR, FB, BM, and OP contributed to the study concept and design, discussed the data analyses. All authors contributed to the article and approved the submitted version.
